# Genes Involved in Oxidative Stress Pathways Are Differentially Expressed in Circulating Mononuclear Cells Derived From Obese Insulin-Resistant and Lean Insulin-Sensitive Individuals Following a Single Mixed-Meal Challenge

**DOI:** 10.3389/fendo.2019.00256

**Published:** 2019-04-24

**Authors:** Sonia Baig, Ehsan Parvaresh Rizi, Chelsea Chia, Muhammad Shabeer, Nweni Aung, Tze Ping Loh, Faidon Magkos, Antonio Vidal-Puig, Raymond C. S. Seet, Chin Meng Khoo, Sue-Anne Toh

**Affiliations:** ^1^Department of Medicine, Yong Loo Lin School of Medicine, National University of Singapore, Singapore, Singapore; ^2^Department of Medicine, National University Health System, Singapore, Singapore; ^3^Trinity College Dublin, University of Dublin, Dublin, Ireland; ^4^Department of Laboratory Medicine, National University Health System, Singapore, Singapore; ^5^Department of Physiology, Yong Loo Lin School of Medicine, National University of Singapore, Singapore, Singapore; ^6^Singapore Institute of Clinical Sciences (SICS), A^*^STAR, Singapore, Singapore; ^7^MRC MDU, Metabolic Research Laboratories, University of Cambridge, Cambridge, United Kingdom; ^8^Programme in Cardiovascular and Metabolic Disorders, Duke-National University of Singapore Graduate Medical School, Singapore, Singapore; ^9^Perelman School of Medicine, University of Pennsylvania, Philadelphia, PA, United States

**Keywords:** obesity, mononuclear cells, oxidative stress, gene expression, macronutrients

## Abstract

**Background:** Oxidative stress induced by nutritional overload has been linked to the pathogenesis of insulin resistance, which is associated with metabolic syndrome, obesity, type 2 diabetes and diabetic vascular complications. Postprandial changes in expression of oxidative stress pathway genes in obese vs. lean individuals, following intake of different types of meals varying in macronutrient composition have not been characterized to date. Here we aimed to test whether/how oxidative stress responses in obese vs. lean individuals are modulated by meal composition.

**Methods:** High-carbohydrate (HC), high-fat (HF), or high-protein (HP) liquid mixed meals were administered to study subjects (lean insulin-sensitive, *n* = 9 and obese insulin-resistant, *n* = 9). Plasma levels of glucose and insulin, lipid profile, urinary F_2_-isoprostanes (F_2_-IsoP), and expression levels of genes of oxidative stress pathways were assessed in mononuclear cells (MNC) derived from fresh peripheral blood, at baseline and up to 6-h postprandial states. Differences in these parameters were compared between insulin-sensitive/resistant groups undergoing aforementioned meal challenges.

**Results:** Obese individuals exhibited increased pro-oxidant (i.e., CYBB and CYBA) and anti-oxidant (i.e., TXN RD1) gene expression in the postprandial state, compared with lean subjects, regardless of meal type (*P* interaction for group × time < 0.05). By contrast, lean subjects had higher expression of NCF-4 gene (pro-oxidant) after HC meal and SOD1 gene (anti-oxidant) after HC and HF meals (*P* interaction for group × meal < 0.05). There was an increase in postprandial level of urinary F_2_-IsoP in the obese (*P* < 0.05) but not lean group.

**Conclusions:** These findings may represent an adaptive oxidative response to mitigate increased stress induced by acute nutritional excess. Further, the results suggest an increased predisposition of obese subjects to oxidative stress. Chronic nutritional excess resulting in increases in body weight and adiposity might lead to decompensation leading to worsening insulin resistance and its sequel. Insights from this study could impact on nutritional recommendations for obese subjects at high-risk of cardiovascular diseases.

## Introduction

Oxidative stress, resulting from an overproduction of oxidants (free radicals or other reactive species) and/or reduced antioxidant activity in cells and plasma, can contribute to impaired insulin signaling (1–4). Oxidative stress occurs early in the development of nutritional excess-induced insulin resistance in healthy men (5, 6). Oxidation of excess nutrients increases mitochondrial formation of reactive oxygen species (ROS) and reactive nitrogen species (RNS) (7). The resultant oxidative stress might induce deleterious changes in macromolecules such as DNA, proteins, and lipids. In addition, a number of stress-sensitive pathways including p38 mitogen-activated protein kinase (p38 MAPK), c-Jun N-terminal kinase (JNK), or inhibitor of NF-κB kinase (IκKβ) are activated (8). These pathways, in turn, impede insulin signaling and glucose transport activity, leading to insulin resistance which is associated with metabolic syndrome, obesity, type 2 diabetes (T2D) and diabetic vascular complications (9). Cumulative perturbations in the regulation of oxidative responses to meal intake, may contribute to the higher risk for atherogenesis and cardiovascular diseases among obese individuals (10).

It is well-known that acute or chronic consumption of a diet rich in (i.e., >50% of caloric composition of) carbohydrate, fat, or protein can worsen the pro-inflammatory and pro-oxidant state associated with obesity, albeit in separate studies (5, 11–15). We have previously shown that the postprandial inflammatory, metabolic and satiety/appetite hormonal responses associated with obesity differ based on the macronutrient content of the meal challenge (16–18). A meal high in carbohydrate, not fat or protein, best elicited these differential responses. Here, we investigated whether the same group of obese insulin resistant individuals demonstrates distinct oxidative stress responses to mixed meals enriched in either of three macronutrients, using both direct (urinary F_2_-isoprostanes) and indirect [expression of genes of oxidative stress pathways in circulating mononuclear cells (MNC)] approaches. Furthermore, we explored if such responses associated with obesity differ with that in lean healthy individuals. Changes in oxidative gene expression profiles in circulating MNC has been previously shown to correspond with that in the adipose tissue in patients with metabolic syndrome (12, 19).

## Materials and Methods

### Study Approval and Subjects

Singapore's National Healthcare Group Domain Specific Review Board (DSRB Ref No: C/2013/00902) approved the study protocol, and Singapore Good Clinical Practice guideline and the principles of the 2013 Declaration of Helsinki were duly followed in performing all study procedures. Written consent was obtained from each subject before participation in this study. The methods have been published before (16–18). Briefly, we recruited 18 normoglycemic Chinese men (21–40 years; lean insulin-sensitive, *n* = 9 and obese insulin-resistant, *n* = 9). Exclusion criteria were history of smoking, thyroid disorder, malignancy, recent hospitalization, or surgery, first degree relative with T2D, dyslipidemia and its treatment, corticosteroids usage over the past 3 months, alcohol consumtion (>3 units a day), moderate-to-high intensity physical activity (>5 h a week), or change in weight over the past 3 months (≥5%). The modified-WHO definition for obesity in Asians was used to define lean (18.5 ≤ BMI ≤ 23 kg/m^2^) and obese (BMI ≥ 27.5 kg/m^2^) subjects in this study. A Homeostatic Model Assessment-Insulin Resistance (HOMA-IR) score of <1.2 was employed for identification of insulin-sensitive lean subjects, and ≥ 2.5 for insulin-resistant obese subjects (20, 21).

### Experimental Design

The experimental design has been described previously (16–18). Briefly, the screening visit included measurements of height, weight and waist circumference, as well as determination of plasma glucose, serum insulin, electrolytes, non-esterified fatty acid (NEFA) concentrations, and lipid profile in fasting blood. Isocaloric liquid mixed meals [high-carbohydrate (HC), high-fat (HF), or high-protein (HP)] were administered to eligible participants in random order with 7 days interval in-between. HC, HF, and HP meals were composed of 56.4% carbohydrate, 56.5% fat [with equal proportions of poly-unsaturated (PUFA), mono-unsaturated (MUFA), and saturated fatty acids (SFA)], and 51.4% protein, respectively (Table S1). Ensure Plus® manufactured by Abbott Nutrition and Beneprotein® manufactured by Nestlé Nutrition were used for preparation of test meals. Baseline and postmeal venous blood samples were collected at 30 min intervals up to 360 min for the measurement of glucose, insulin, triglyceride and NEFA concentrations. Fasting and postprandial (360 min) midstream urine samples were also collected for the measurement of urinary F_2_-isoprostanes, a biomarker of oxidative stress -induced lipid peroxidation (22, 23) to assess systemic oxidative stress.

### Biochemical Analysis

Measurements of plasma glucose and triglyceride concentrations (AU5800, Beckman Coulter Inc., California, USA), and serum insulin (ADVIA Centaur, Siemens Healthcare Diagnostics, Hamburg, Germany) were performed at a laboratory accredited by the College of American Pathologists. Measurement of plasma NEFA (Cobas® 6000, Roche Diagnostics, Indianapolis, USA) was performed at Mayo Medical Laboratories (Rochester, MN, USA). Urinary free F_2_-isoprostanes were measured using a method described previously (23, 24). Briefly, urine samples were processed by anionic solid-phase extraction. Creatinine levels were measured to standardize the dilution of urine (Cobas c111 Photometric Analyzer (Roche Diagnostic GmbH, Mannheim, Germany). Samples were then derivatized and measured by gas chromatography–mass spectrometry (GC/MS) set at negative chemical ionization mode (5975C; Agilent Technologies), with Triple-Axis Detector, connected to a gas chromatograph (7890A; Agilent Technologies, Santa Clara, CA). Quantitation was achieved by comparing the peak area of free F_2_-isoprostanes with that of the relevant deuterated internal standard.

### Gene Expression

Blood samples collected at 0, 120, and 360 min, were layered over Ficoll-paque Plus (GE Healthcare, Buckinghamshire, UK) and centrifuged. Following red blood cell lysis (Sigma-Aldrich, St. Louis, MO, USA), total RNA from MNC was isolated using RNeasy Mini Kit (QIAGEN, Netherlands). For reverse transcription of total RNA, high capacity cDNA Reverse-Transcription Kit (Applied Biosystems, Waltham, MA, USA) was used. ViiA 7 Real-Time PCR System (Applied Biosystems) was used to perform gene expression assay. The PCR mix included 2 μL (10 ng) cDNA, 5 μL QuantiFast SYBR Green PCR Master mix (QIAGEN, Netherlands), and 0.1 μL of 100 μmol/L gene-specific primers (AIT Biotech, Singapore). Primers were designed using Primer Express software v3.0.1 (Applied Biosystems). All values were normalized to the expression of a housekeeping gene (GAPDH), which did not differ among the different phenotypes, time points and types of test meal. The panel of genes studied included, Nuclear factor, erythroid 2-like 2 (NRF2), Glutathione peroxidase (GPX3), Thioredoxin (TXN), Thioredoxin reductase 1 (TXNRD1), Superoxide dismutase (SOD- 1 and -2), Human neutrophil cytochrome –A light chain and –B light chain (CYBA and CYBB), Neutrophil cytosolic factor (NCF-1,-2, and -4), and Spi-1 (PU.1). Three sets of samples (2 lean subjects, 1 obese subject) were excluded from analysis due to poor quality of RNA.

### Statistical Analysis

The primary outcome of the original study which was designed to assess postprandial inflammatory responses, was fold changes in expression of inflammatory genes (regulated by NF-κB) in MNC, from baseline as an indicator of NF-κB activity. Power analysis was based on the postmeal NF-κB expression, whereby a sample size of 9 subjects per group per test meal was calculated to provide at least 80% power at 5.0% significance level (25).

Statistical analyses were performed using SPSS version 23.0 (SPSS Inc., Chicago, IL, USA). A linear mixed model was employed to analyse MNC gene expression between groups and meals. Fold-change from baseline in gene expression MNC was entered as the dependent variable, while time and meal were entered as repeated factors. Change in the trajectories of gene expression was further tested for interaction. Linear model with fixed effects for meal and individual was used to test whether postprandial changes in urinary F_2_-IsoP was significant in obese and lean group. An independent sample *t*-test was used to test the differences in fold-changes in MNC gene expression at a single time point between groups. Postprandial changes in plasma glucose and insulin concentrations over 6 h were calculated as the incremental area under the curve (iAUC). Fold changes in expression of genes were tested for a significant correlation with glucose and insulin iAUC. A value of *P* < 0.05 was considered statistically significant.

## Results

### Subject Characteristics at Baseline

Obese subjects had higher age (obese: 28.6 ± 1.4_year_ vs. lean: 23.2 ± 0.2_year_; *P* = 0.002), body mass index (obese: 30.1 ± $0.7_{\text{kg}/\text{m}}^{2}$ vs. lean: 22.0 ± $0.2_{\text{kg}/\text{m}}^{2}$; *P*_*age adjusted*_ < 0.001), waist circumference (obese: 100.8 ± 0.1_cm_ vs. lean: 79.9 ± 0.5_cm_; *P*_*age adjusted*_ < 0.001), HOMA-IR (obese: 4.3 ± 0.4 vs. lean: 0.8 ± 0.1; *P*_*age adjusted*_ < 0.001), fasting serum insulin (obese: 21.0 ± 2.3_mU/l_ vs. lean: 4.3 ± 0.5_mU/l_; *P*_*age adjusted*_ < 0.001) and plasma triglyceride concentrations (obese: 2.0 ± 0.2_mmol/l_ vs. lean: 0.6 ± 0.1_mmol/l_; *P*_*age adjusted*_ = 0.007), and lower HDL-cholesterol concentration (obese: 1.2 ± 0.1_mmol/l_ vs. lean: 1.7 ± 0.1_mmol/l_; *P*_*age adjusted*_ = 0.005) compared to lean subjects (Supplementary Data Sheet 1). Fasting blood glucose, total and LDL cholesterol and NEFA were not statistically different between groups (16–18).

### Postprandial Changes in Glucose, Insulin, Triglyceride and NEFA Responses

Overall, the postprandial insulin and triglycerides levels increased to a higher level in the obese than lean subjects while postprandial glucose responses were similar between the two groups. These data have been reported previously in detail (16–18).

### Postprandial Changes in Expression of NADPH-oxidase Genes

**Figure 1:**
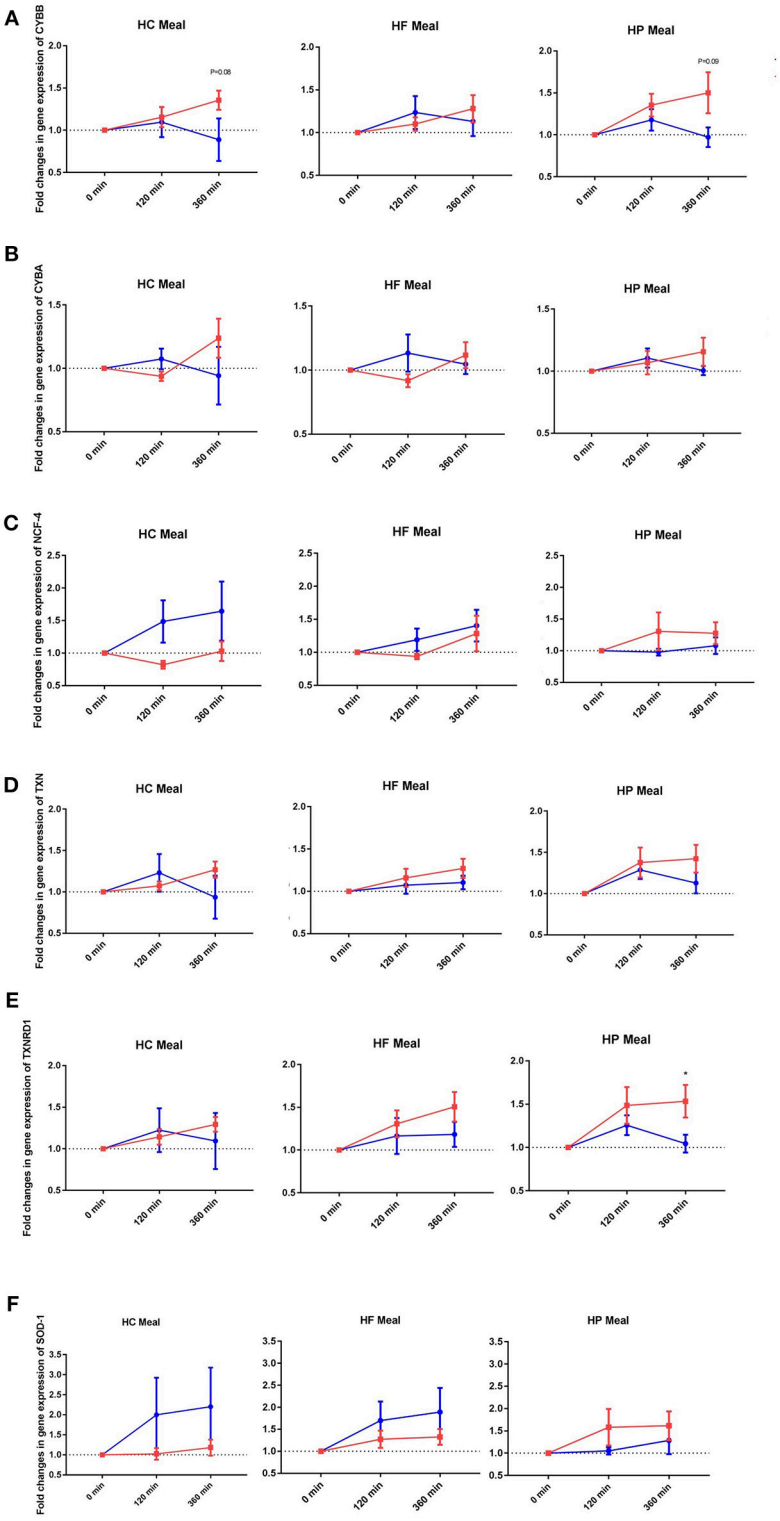
Fold changes from baseline in expression of **(A)** CYBB, **(B)** CYBA, **(C)** NCF-4, **(D)** TXN, **(E)** TXNRD1, and **(F)** SOD-1 genes in MNC between lean insulin-sensitive (Blue,•) and obese insulin-resistant (Red,■) individuals, following consumption of isoenergetic liquid mixed meals. Values are mean ± SEM. Single time point comparisons between two groups, by using unpaired *t-*test, are indicated with (^*^) when significant (*P* < 0.05).

NADPH-oxidases constitute an enzyme complex at cell membrane that produces superoxide, a substrate for subsequent reactions to generate ROS. Mean postprandial fold changes for gene expression of NADPH-oxidase subunits (CYBA and CYBB; NCF-1,-2, and -4) were not significant between groups, meals or single time points (Table 1, Supplementary Data Sheet 2). However, CYBB and CYBA gene expression increased over 6 h in obese than lean subjects, irrespective of meal type (*P* interaction for group × time < 0.05) (Table 1, Figures 1A,B). Conversely, obese subjects had lower expression of NCF-4 gene compared to lean subjects after the HC meal (*P* interaction for group × meal < 0.05) (Table 1, Figure 1C).

**Table 1:** Fold changes in MNC gene expression in lean and obese subjects 2 and 6 h after consuming 3 isocaloric liquid mixed meals.

		**HC meal**		**HF meal**		**HP meal**		**Main effects**			**Interaction effects**		
		**Δ 2 h**	**Δ 6 h**	**Δ 2 h**	**Δ 6 h**	**Δ 2 h**	**Δ 6 h**	**Group**	**Time**	**Meal**	**Group × Meal**	**Group × Time**	**Meal × Time**
CYBB	LIS	1.10 ± 0.18	0.89 ± 0.25	1.23 ± 0.19	1.13 ± 0.17	1.18 ± 0.13	0.97 ± 0.12	0.132	**0.005**	0.309	0.202	**0.007**	0.839
	OIR	1.15 ± 0.12	1.35 ± 0.11	1.10 ± 0.08	1.28 ± 0.16	1.35 ± 0.13	1.50 ± 0.24						
CYBA	LIS	1.07 ± 0.08	0.94 ± 0.23	1.13 ± 0.14	1.05 ± 0.08	1.11 ± 0.08	1.00 ± 0.04	0.758	0.167	0.870	0.446	**0.006**	0.845
	OIR	0.94 ± 0.04	1.24 ± 0.15	0.92 ± 0.05	1.12 ± 0.10	1.07 ± 0.09	1.16 ± 0.11						
NCF1	LIS	1.12 ± 0.12	1.10 ± 0.16	1.14 ± 0.12	1.41 ± 0.27	1.01 ± 0.06	1.15 ± 0.10	0.350	**0.002**	0.416	0.182	0.730	0.811
	OIR	0.88 ± 0.07	1.09 ± 0.14	0.97 ± 0.06	1.15 ± 0.15	1.11 ± 0.12	1.26 ± 0.13						
NCF2	LIS	1.09 ± 0.10	1.18 ± 0.18	1.15 ± 0.13	1.33 ± 0.18	1.06 ± 0.06	1.03 ± 0.09	0.099	**0.017**	0.495	0.088	0.348	0.761
	OIR	0.92 ± 0.07	1.01 ± 0.11	0.95 ± 0.05	1.08 ± 0.12	1.06 ± 0.08	1.14 ± 0.07						
NCF4	LIS	1.49 ± 0.32	1.64 ± 0.45	1.19 ± 0.17	1.40 ± 0.24	0.98 ± 0.05	1.08 ± 0.13	0.229	**0.007**	0.920	**0.008**	0.573	0.804
	OIR	0.82 ± 0.06	1.03 ± 0.15	0.94 ± 0.04	1.28 ± 0.27	1.31 ± 0.30	1.28 ± 0.17						
SOD1	LIS	1.20 ± 0.93	2.20 ± 0.97	1.70 ± 0.43	1.89 ± 0.55	1.05 ± 0.08	1.29 ± 0.31	0.288	**0.001**	0.751	**0.023**	0.503	0.992
	OIR	1.02 ± 0.14	1.18 ± 0.20	1.27 ± 0.19	1.33 ± 0.18	1.58 ± 0.41	1.62 ± 0.32						
SOD2	LIS	1,20 ± 0.22	1.22 ± 0.33	1.14 ± 0.15	1.37 ± 0.21	1.02 ± 0.05	1.09 ± 0.08	0.932	**0.005**	0.350	0.252	0.822	0.388
	OIR	0.99 ± 0.12	1.00 ± 0.22	1.04 ± 0.06	1.45 ± 0.24	1.15 ± 0.15	1.33 ± 0.15						
GPx3	LIS	1.25 ± 0.12	1.29 ± 0.30	1.22 ± 0.12	1.31 ± 0.11	1.17 ± 0.06	1.28 ± 0.25	0.560	**0.001**	0.670	0.859	0.534	0.607
	OIR	1.13 ± 0.07	1.70 ± 0.41	1.18 ± 0.13	1.43 ± 0.13	1.24 ± 0.16	1.23 ± 0.15						
TXN	LIS	1.23 ± 0.23	0.94 ± 0.26	1.07 ± 0.10	1.10 ± 0.08	1.29 ± 0.11	1.13 ± 0.13	0.161	**0.001**	0.059	0.859	0.057	0.435
	OIR	1.07 ± 0.05	1.27 ± 0.10	1.16 ± 0.11	1.27 ± 0.11	1.38 ± 0.18	1.42 ± 0.17						
TXNRD1	LIS	1.22 ± 0.26	1.09 ± 0.34	1.16 ± 0.21	1.18 ± 0.15	1.26 ± 0.11	1.04 ± 0.10	0.096	**0.001**	0.218	0.446	**0.046**	0.588
	OIR	1.14 ± 0.09	1.29 ± 0.09	1.31 ± 0.16	1.51 ± 0.17	1.49 ± 0.21	1.53 ± 0.19						
Nrf2	LIS	1.32 ± 0.29	1.42 ± 0.35	1.16 ± 0.14	1.40 ± 0.18	1.06 ± 0.06	1.12 ± 0.09	0.874	**0.001**	0.619	0.137	0.975	0.834
	OIR	1.17 ± 0.10	1.32 ± 0.11	1.12 ± 0.09	1.28 ± 0.10	1.29 ± 0.16	1.34 ± 0.13						

*Significant values (P < 0.05) are indicated in bold*.

### Postprandial Changes in Gene Expression of Antioxidant Redox-Related Proteins

TXN is a small redox protein and TXNRD1 is the enzyme that reduces TXN from the oxidized to the reduced, active form for neutralization of ROS. Mean postprandial fold changes for expression of the aforementioned genes were not significant between groups, meals or single time points (Table 1, Figures 1D,E, Supplementary Data Sheet 2). Of note, TXNRD1 gene showed higher increase over 6 h in obese compared to lean subjects irrespective of meal type (*P* interaction for group × time < 0.05) (Table 1, Figure 1E). This may represent an adaptive response to counter the upregulation of NADPH-oxidases in the obese individuals, and thus achieve redox homeostasis.

### Postprandial Changes in Antioxidant Enzyme Genes

We also examined gene expression of antioxidant enzymes (SOD1, SOD2, and GPX3), which showed modest increases in both groups following all test meals (*P* for time effect < 0.05) (Table 1, Supplementary Data Sheet 2). Mean postprandial fold changes for expression of the aforementioned genes were not significant between groups, meals or single time points. Of relevance, obese subjects had lower increase in SOD1 after HC and HF meals compared to lean subjects (*P* interaction for group × meal < 0.05) (Table 1, Figure 1F).

### Postprandial Changes in Antioxidant Response Regulatory Genes

Mean postprnadial fold changes for expression in NRF2 was not significant between groups, meals or single time points (Table 1, Supplementary Data Sheet 2). The NRF2 transcription factor is an antioxidant response regulatory transcription factor, and an increase in its expression in nucleus indicates oxidative damage at the cellular level. In the cytosol, it is bound to Keap1 and remains in an inactivated state. Upon cellular encounter with stress, the Keap1-NRF2 complex undergoes disruption and NRF2 is transferred to the nucleus.

### Postprandial Changes in Urinary Free F_2_-Isoprostanes (F_2_-IsoPs)

**Figure 2:**
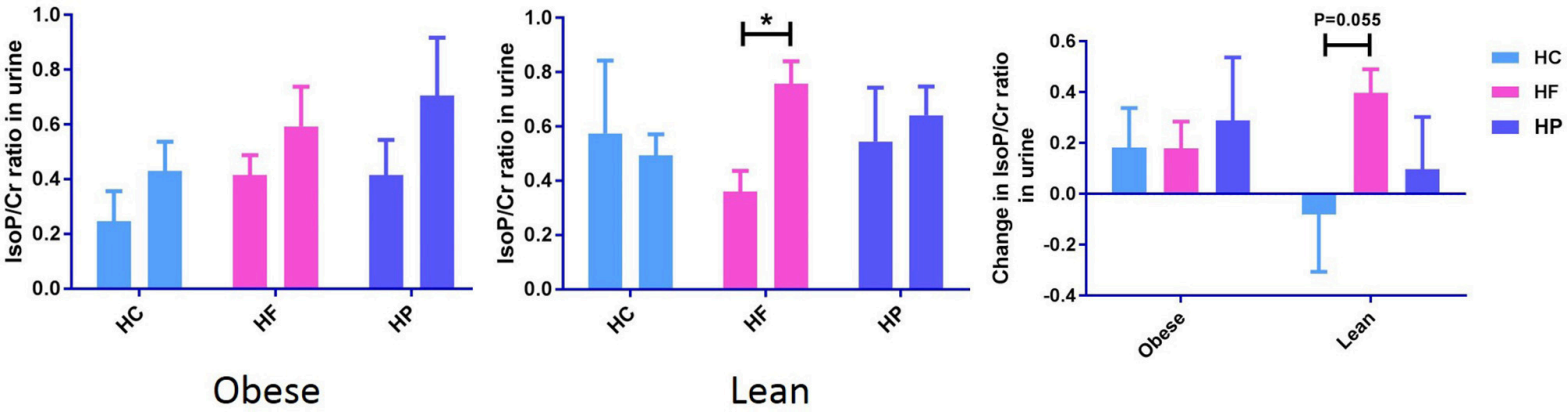
Postprandial change in urine F_2_-IsoPs concentration corrected for urine creatinine concentration (F_2_-IsoP/Cr ratio) from baseline to 6 h after high carbohydrate (HC), high-fat (HF), and high-protein (HP) meal ingestion in lean and obese individuals. Values are mean ± SEM and analyzed by using two-tailed *t*-tests between lean vs. obese subject after HC (−0.08 ± 0.23 vs. 0.18 ± 0.15, *P* = 0.35), HF (0.40 ± 0.09 vs. 0.18 ± 0.11, *P* = 0.14), and HP (0.10 ± 0.21 vs. 0.29 ± 0.25, *P* = 0.56) meals, and indicated with (^*^) when significant (*P* < 0.05). Independent of diet, postprandial urinary F_2_-IsoP/Cr significantly increased in obese, but not lean individuals.

Urinary creatinine (Cr) levels, measured to standardize the dilution of urine, did not differ between the different dietary interventions. Independent of diet, postprandial urinary F_2_-IsoP/Cr significantly increased in obese (*P* < 0.05), but not lean individuals (Supplementary Data Sheet 3). The paired sample *t*-test also revealed that in lean subjects, postprandial change in urinary F_2_-IsoP/Cr from baseline to 6 h, differs between HC and HF meal (*P* = 0.055) (Figure 2). F_2_-IsoPs are prostaglandin (PG) F_2_-like compounds. These are produced as a result of free radical catalyzed peroxidation of arachidonic acid and are currently considered the gold standard among markers of systemic oxidative damage.

### Correlation Analysis

We analyzed the relationship between postprandial fold changes in MNC gene expression (at 120 and 360 min) vs. iAUC of serum insulin and plasma glucose (Table S2). Insulin iAUC correlated with increased fold changes in expression of CYBB (*r*: 0.42; *P* = 0.07), CYBA (*r*: 0.51; *P* = 0.03), and TXN (*r*: 0.41; *P* = 0.08) genes in the obese MNC at 120 min following meal ingestion. Glucose iAUC correlated with increased fold changes in expression of NCF1 (*r*: 0.52; *P* = 0.07), NCF4 (*r*: 0.52; *P* = 0.07) and SOD1(*r*: 0.49; *P* = 0.09) genes at 120 min, and NCF2 (*r*: 0.52; *P* = 0.07), NCF4 (*r*: 0.54; *P* = 0.06), SOD1 (*r*: 0.57; *P* = 0.04), and SOD2 (*r*: 0.55; *P* = 0.05) genes at 360 min in lean MNC after meal ingestion.

## Discussion

In this study, we compared expression of genes of oxidative stress pathways in MNC following intake of HC, HF, and HP meals in a metabolically distinct cohort of lean insulin-sensitive and obese insulin-resistant individuals (with hypertriglyceridemia). We found that the individual's underlying metabolic phenotype has a differential impact on oxidative gene expression in circulating MNCs. This was evident based on differences in the direction and magnitude of changes seen in the postprandial oxidative gene expression profiles in MNC as well as systemic oxidative stress marker F_2_-IsoP in urine, over the postprandial period between the two groups.

The overall trend toward higher expression of the pro-oxidant genes involved in the oxidative pathway in both obese and lean groups may indirectly reflect a physiological increase in ROS generation in the postprandial state. However, we found that the expression of anti-oxidant group of genes were also elevated suggesting an adaptive response to mitigate the higher postprandial oxidative stress among the study participants. Our findings are in concordance with that by Camargo et al. who reported an increase in the postprandial expression of both pro- and anti-oxidant genes in the MNC of individuals with metabolic syndrome in response to a 12-week HF diet (12). Likewise, in another study, Patel et al. showed that a single HF-HC meal challenge induced oxidative and inflammatory stress responses greater both in magnitude and duration, as evident by increases in the expression of NCF-1 (a major ROS-generating enzyme), intracellular NF-κB binding activity and plasma concentrations of MMP-9, in the MNCs in obese compared to lean individuals (11). Of note, in the current study, there were consistent trends toward greater duration and magnitude of oxidative responses in obese individuals (with hypertriglyceridemia) following HC and HP meals compared to HF meal, suggesting an increased predisposition of these subjects to oxidative stress.

In the current study, expressions of CYBB and CYBA (catalytic parts of NADPH oxidase) genes increased over 6-h following meal consumption in obese compared to lean patients, while changes in expression of NCF-4 (cytosolic activator of NADPH oxidase) gene were the contrary. The opposite direction of postprandial changes in NCF-4 expression following intake of HC and HF vs. HP meal, led to a significant group x meal interaction. The trend toward lower NCF-4 expression in obese group may be explained as a protective negative feedback phenomenon exerted by existing exaggerated oxidative stress associated with obesity. It is known that enhanced production of reactive oxygen or nitrogen species due to augmented NADPH oxidase activity and ER stress in adipose tissue characterizes obesity (26, 27). Further, antioxidant defenses are lower in obese compared to that in lean individuals (28, 29).

NRF2 is a nuclear transcription factor and its activation can be characterized by protein expression assay, i.e., western blotting using nuclear component of freshly isolated cells, i.e., MNC in this case. Since fresh MNC were not available at the time of this oxidative stress response study for protein isolation, the protein expression levels of NRF2 could not be assessed. An upregulation in its expression at gene level could not be observed alongside the increases in expression of several anti-oxidant genes in the current study, as would have been expected. However, the transcription level data can only suggest whether the protein is present and approximately its expected level and needs to be validated by western blot assay.

F_2_-isoprostanes, are accurate indicators of systemic oxidative stress *in vivo* (23), and showed trends similar to those observed for MNC gene expression. We observed an increase in postprandial urinary F_2_-IsoP/Cr in the obese group (*P* < 0.05), independent of meal type. These results may suggest an increased predisposition of obese subjects (with hypertriglyceridemia) to oxidative stress. Camargo et al. found positive correlations between plasma levels of oxidative stress markers such as protein carbonyl, H_2_O_2_, etc. and expression of genes of oxidative pathway in obese-derived MNC, 2-h after meal intake (12). Although the marker of systemic oxidative stress assessed in the current study is different from those in the aforementioned study, the postprandial changes we observed in the obese, are similar to theirs. Interestingly, significant postprandial increases in urinary F_2_-IsoP level could be observed in lean individuals following HF meal and the changes were near-significantly higher than that following HC meal. This could be attributed to the fact that the obese subjects are “adapted” to a diet high in fat, and so do not experience the same systemic oxidative stress response to a diet high in fat as the lean group. Conversely, the lean group may be adapted to a more carbohydrate/protein rich diet.

It is well known that MUFA rich diet exerts an anti-inflammatory, antioxidant effect (30–32). Despite an equal proportion of MUFA, PUFA, and SFA in the HF meal in this study, we only observed a modest/minimal effect (on postprandial gene expression as well as urinary F_2_-isoprostanes) exerted by HF meal. The total caloric content as well as SFA proportion was much higher in previous studies as compared to that in our study (11, 32, 33). The deficiency/lack of relative and absolute amount of SFA in the HF meal could have contributed to the more modest changes observed in our study, both in postprandial MNC gene expression and systemic F_2_-Isop responses (34).

We observed positive correlations between the postprandial serum insulin response with expression of pro- and antioxidant genes in the obese insulin resistant individuals (with hypertriglyceridemia). The obese subjects had significant postprandial hyperinsulinemia compared to the lean group despite similar glycemic response, indicative that they require more insulin to maintain the same glucose tolerance owing to peripheral tissue insulin resistance. Our findings are in agreement with that of Patel et al who demonstrated significantly higher insulin levels, alongside higher expression of oxidative stress markers in the MNCs of obese individuals in the postprandial state (11). These results support the emerging notion that oxidative stress is among the key events leading to insulin resistance, which is pivotal in the pathogenesis and progression of T2D (9, 35, 36). An increase in the mitochondrial ROS generation from a nutrient-rich environment induces cellular stress pathways resulting in insulin resistance by interrupting insulin receptor signal transduction. We propose that it represents a check-and-balance response in the obese individuals such that the expression of anti-oxidant genes increased in tandem to the pro-oxidant genes regardless of the macronutrient content.

Strengths of our study include the fact that we compared two distinct metabolic phenotypes, i.e., lean insulin-sensitive and obese insulin-resistant individuals (with hypertriglyceridemia) and that within each group, the individuals were homogeneous. In addition, we compared three test meals of different macronutrient composition in the same group of subjects. The caloric contents of these test meals were well representative of normal dietary intake. By contrast, previous studies examined only a single phenotype in isolation (either metabolic syndrome or non-obese individuals), following intake of one type of meal (mostly HF, not representative of normal dietary intake) (5, 6, 12–15). To our understanding, postprandial changes in expression of oxidative stress pathway genes have not been assessed previously, following intake of different types of isocaloric mixed- meals enriched in either of all three major macronutrients (carbohydrate, fat, or protein), in a cohort both obese insulin-resistant and lean insulin-sensitive individuals. Interestingly, our previous work on the same cohort has shown differential postprandial inflammatory, metabolic as well as satiety/appetite hormonal response (16–18).

However, these findings, researched using a gene expression approach, could be further validated with protein expression studies. We acknowledge postprandial changes in gene expression in MNC are indeed among indirect measures of oxidative stress, while that in urinary isoprostane level are among direct measures. Since fresh MNC were not available for further analysis, protein expression levels and additional assays to measure intracellular oxidative stress such as reduced/oxidized glutathione level, could not be assessed. These direct measures of oxidative stress have since been incorporated in our subsequent/ongoing studies in other metabolic risk phenotypes such as individuals with heredity of type 2 diabetes, prediabetes, etc.

Further, we would like to highlight that our study subjects of interest are otherwise healthy, normoglycemic individuals, who are capable of adaptive/compensatory response in postprandial state to achieve homeostasis. Perhaps this may be why we have seen significant changes in expression of some, but not all genes. The direction and magnitude of changes in the measured parameters trended to be different between the two groups, despite being otherwise healthy and normoglycemic. Thus profiling of postprandial gene expression levels could be a potential early marker for monitoring progression/worsening of metabolic disorders long before conventional clinical markers demonstrate appreciable changes.

To conclude, acute nutritional intake may lead to oxidative stress followed by an adaptive, compensatory response in order to mitigate postprandial stress. However, chronic nutritional excess resulting in weight gain and increased adiposity may lead to decompensation and in turn, worsen insulin resistance and its sequelae. Our findings support an increased predisposition of obese subjects (with hypertriglyceridemia) to oxidative stress, particularly in response to a meal rich in carbohydrate or protein.

## Ethics Statement

This study was carried out in accordance with the recommendations of Singapore's National Healthcare Group Domain Specific Review Board (DSRB Ref No: C/2013/00902), and all procedures followed the Singapore Good Clinical Practice guideline and the principles of the 2013 Declaration of Helsinki. All subjects gave written informed consent in accordance with the Declaration of Helsinki. The protocol was approved by Singapore's National Healthcare Group Domain Specific Review Board.

## Author Contributions

SB contributed to planning and execution of wet laboratory experiments, acquisition, analyses and interpretation of data from these experiments, prepared the first draft of the manuscript and secured funding. EP contributed to planning and execution of physiology experiments, acquisition, analyses and interpretation of data from these experiments, edited the manuscript and secured funding. CC, MS, and NA performed MNC isolation, the gene expression assay and edited the manuscript. TL, AV-P, FM, and RS contributed to study design, grant proposal and critically revised the manuscript. CK and S-AT contributed to study conception and design, data interpretation and critical revision of the manuscript.

### Conflict of Interest Statement

The authors declare that the research was conducted in the absence of any commercial or financial relationships that could be construed as a potential conflict of interest. The reviewer DF declared a shared affiliation, with no collaboration, with one of the authors AV to the handling Editor.

## Supplementary Material

Supplementary Data Sheet 1 Baseline Values Database.

Supplementary Data Sheet 2 Gene Expression Database.

Supplementary Data Sheet 3 F2-isoprostane Database.
