# Supplementary material for: Genes Involved in Oxidative Stress Pathways Are Differentially Expressed in Circulating Mononuclear Cells Derived From Obese Insulin-Resistant and Lean Insulin-Sensitive Individuals Following a Single Mixed-Meal Challenge
Source: Front Endocrinol (Lausanne). 2019 Apr 24;10:256. doi: 10.3389/fendo.2019.00256 (PMC6491694; doi:10.3389/fendo.2019.00256)
Supplement: Supplementary file 1 [file Table_1.DOCX]

**Table S1.** Macronutrient composition of the 3 different liquid mixed meals

|  |  | **Energy**  **(Kcal)** | **Carb.**  **(g)** | **Protein**  **(g)** | **Fat**  **(g)** | **MUFA**  **(g)** | **PUFA**  **(g)** | **SFA**  **(g)** |
| --- | --- | --- | --- | --- | --- | --- | --- | --- |
| **HF Meal** | Total | **598.34** | 52.00 | 13.31 | 37.57 | 12.24 | 11.54 | 11.81 |
|  | % composition |  |  |  |  | 32.6 | 30.7 | 31.4 |
|  | % Kcal |  | 34.8 | 8.9 | **56.5** |  |  |  |
| **HC Meal** | Total | **599.25** | 84.49 | 21.25 | 19.13 | 4.65 | 11.28 | 2.42 |
|  | % composition |  |  |  |  | 24.3 | 59.0 | 12.7 |
|  | % Kcal |  | **56.4** | 14.2 | 28.7 |  |  |  |
| **HP Meal** | Total | **599.19** | 45.72 | 76.95 | 10.35 | 2.52 | 6.11 | 1.31 |
|  | % composition |  |  |  |  | 24.3 | 59.0 | 12.7 |
|  | % Kcal |  | 30.5 | **51.4** | 15.5 |  |  |  |

High carbohydrate: HC, high fat: HF, high protein: HP, MUFA: monounsaturated fatty acids, PUFA: polyunsaturated fatty acids, SFA: saturated fatty
