# Supplementary material for: Genes Involved in Oxidative Stress Pathways Are Differentially Expressed in Circulating Mononuclear Cells Derived From Obese Insulin-Resistant and Lean Insulin-Sensitive Individuals Following a Single Mixed-Meal Challenge
Source: Front Endocrinol (Lausanne). 2019 Apr 24;10:256. doi: 10.3389/fendo.2019.00256 (PMC6491694; doi:10.3389/fendo.2019.00256)
Supplement: Supplementary file 2 [file Table_2.DOCX]

**Table S2.** Within subjects correlation between postprandial fold changes in expression of genes and iAUC for plasma glucose and serum insulin over 6-h after the meal

|  |  |  | 6-h insulin iAUC | | 6-h glucose iAUC | |
| --- | --- | --- | --- | --- | --- | --- |
|  |  |  | r | *P* value | r | *P* value |
| CYBB | 120 min. | Lean | -0.12 | 0.72 | -0.41 | 0.20 |
|  |  | Obese | **0.42** | **0.07** | -0.06 | 0.82 |
|  |  | Total | **0.36** | **0.05** | -0.14 | 0.48 |
|  | 360 min. | Lean | -0.32 | 0.32 | 0.17 | 0.60 |
|  |  | Obese | 0.26 | 0.28 | -0.16 | 0.52 |
|  |  | Total | 0.21 | 0.27 | -0.08 | 0.69 |
| CYBA | 120 min. | Lean | -0.40 | 0.21 | -0.13 | 0.70 |
|  |  | Obese | **0.51** | **0.03** | -0.37 | 0.13 |
|  |  | Total | **0.35** | **0.07** | -0.28 | 0.14 |
|  | 360 min. | Lean | -0.26 | 0.43 | 0.08 | 0.81 |
|  |  | Obese | 0.14 | 0.57 | -0.17 | 0.49 |
|  |  | Total | 0.09 | 0.65 | -0.09 | 0.64 |
| NCF-1 | 120 min. | Lean | -0.05 | 0.88 | 0.52 | 0.07 |
|  |  | Obese | 0.33 | 0.17 | -0.29 | 0.23 |
|  |  | Total | 0.25 | 0.19 | 0.01 | 0.96 |
|  | 360 min. | Lean | 0.02 | 0.96 | 0.32 | 0.30 |
|  |  | Obese | 0.06 | 0.81 | -0.32 | 0.19 |
|  |  | Total | 0.05 | 0.79 | -0.07 | 0.70 |
| NCF-2 | 120 min. | Lean | 0.05 | 0.86 | 0.46 | 0.12 |
|  |  | Obese | 0.34 | 0.16 | -0.16 | 0.52 |
|  |  | Total | 0.25 | 0.19 | 0.11 | 0.58 |
|  | 360 min. | Lean | -0.01 | 0.97 | 0.52 | 0.07 |
|  |  | Obese | 0.07 | 0.79 | -0.39 | 0.10 |
|  |  | Total | 0.04 | 0.82 | 0.03 | 0.87 |

|  |  |  | 6-h insulin iAUC | | 6-h glucose iAUC | |
| --- | --- | --- | --- | --- | --- | --- |
|  |  |  | r | *P* value | r | *P* value |
| NCF-4 | 120 min. | Lean | -0.12 | 0.71 | 0.52 | 0.07 |
|  |  | Obese | 0.40 | 0.09 | -0.26 | 0.29 |
|  |  | Total | 0.29 | 0.12 | 0.02 | 0.90 |
|  | 360 min. | Lean | -0.03 | 0.92 | 0.54 | 0.06 |
|  |  | Obese | 0.03 | 0.91 | -0.31 | 0.21 |
|  |  | Total | 0.01 | 0.94 | 0.07 | 0.70 |
| SOD1 | 120 min. | Lean | -0.10 | 0.75 | 0.49 | 0.09 |
|  |  | Obese | 0.36 | 0.14 | -0.27 | 0.27 |
|  |  | Total | 0.19 | 0.31 | 0.10 | 0.59 |
|  | 360 min. | Lean | 0.00 | 0.99 | 0.57 | 0.04 |
|  |  | Obese | 0.33 | 0.17 | -0.15 | 0.54 |

|  |  | Total | 0.11 | 0.58 | 0.31 | 0.10 |
| --- | --- | --- | --- | --- | --- | --- |
| SOD2 | 120 min. | Lean | -0.02 | 0.96 | 0.38 | 0.21 |
|  |  | Obese | 0.28 | 0.25 | -0.20 | 0.42 |
|  |  | Total | 0.20 | 0.28 | 0.03 | 0.87 |
|  | 360 min. | Lean | -0.02 | 0.94 | 0.55 | 0.05 |
|  |  | Obese | -0.11 | 0.66 | -0.22 | 0.37 |
|  |  | Total | -0.10 | 0.61 | 0.02 | 0.92 |
| GPx3 | 120 min. | Lean | -0.19 | 0.57 | 0.38 | 0.24 |
|  |  | Obese | 0.13 | 0.61 | -0.19 | 0.44 |
|  |  | Total | 0.09 | 0.66 | -0.03 | 0.88 |
|  | 360 min. | Lean | -0.08 | 0.80 | 0.08 | 0.80 |
|  |  | Obese | -0.20 | 0.42 | 0.04 | 0.88 |
|  |  | Total | -0.17 | 0.38 | 0.05 | 0.78 |

|  |  |  | 6-h insulin iAUC | | 6-h glucose iAUC | |
| --- | --- | --- | --- | --- | --- | --- |
|  |  |  | r | *P* value | r | *P* value |
| TXN | 120 min. | Lean | 0.36 | 0.27 | -0.06 | 0.86 |
|  |  | Obese | **0.41** | **0.08** | -0.20 | 0.42 |
|  |  | Total | **0.39** | **0.04** | -0.15 | 0.44 |
|  | 360 min. | Lean | -0.33 | 0.30 | 0.04 | 0.92 |
|  |  | Obese | 0.37 | 0.12 | -0.13 | 0.60 |
|  |  | Total | 0.26 | 0.17 | -0.07 | 0.70 |
| TXN RD | 120 min. | Lean | -0.14 | 0.67 | -0.49 | 0.11 |
|  |  | Obese | 0.31 | 0.21 | -0.16 | 0.53 |
|  |  | Total | 0.27 | 0.16 | -0.21 | 0.28 |
|  | 360 min. | Lean | -0.53 | 0.08 | 0.17 | 0.60 |
|  |  | Obese | 0.18 | 0.46 | -0.20 | 0.43 |

|  |  | Total | 0.09 | 0.65 | -0.07 | 0.70 |
| --- | --- | --- | --- | --- | --- | --- |
| Nrf2 | 120 min. | Lean | 0.00 | 1.00 | 0.39 | 0.19 |
|  |  | Obese | 0.32 | 0.20 | -0.13 | 0.62 |
|  |  | Total | 0.23 | 0.24 | 0.09 | 0.63 |
|  | 360 min. | Lean | -0.01 | 0.98 | 0.39 | 0.19 |
|  |  | Obese | 0.03 | 0.91 | -0.19 | 0.44 |
|  |  | Total | 0.02 | 0.92 | 0.09 | 0.65 |
